# Advances in Stabilizing Spinel Cobalt Oxide‐Based Catalysts for Acidic Oxygen Evolution Reaction

**DOI:** 10.1002/advs.202509415

**Published:** 2025-07-28

**Authors:** Chengli Rong, Qian Sun, Jiexin Zhu, Hamidreza Arandiyan, Zongping Shao, Yuan Wang, Yuan Chen

**Affiliations:** ^1^ School of Chemical and Biomolecular Engineering The University of Sydney Darlington New South Wales 2006 Australia; ^2^ Department of Mechanical and Industrial Engineering University of Toronto Toronto Ontario M5S 3G8 Canada; ^3^ Centre for Advanced Materials and Industrial Chemistry (CAMIC) School of Science RMIT University Melbourne VIC 3000 Australia; ^4^ WA School of Mines: Minerals Energy and Chemical Engineering Curtin University Perth WA 6845 Australia; ^5^ Department of Chemical Engineering The University of Melbourne VIC 3010 Australia

**Keywords:** catalyst stability, oxygen evolution reaction, spinel Co_3_O_4_ catalyst

## Abstract

Oxygen evolution reaction (OER) is pivotal to sustainable energy storage and conversion technologies. Yet, its sluggish kinetics in acidic media and reliance on expensive noble metal catalysts limit its efficiency in these applications. Spinel cobalt(II, III) oxide (Co_3_O_4_)‐based catalysts are cost‐effective alternatives with high theoretical catalytic activity. However, their practical deployment is hindered by their poor stability in acidic electrolytes. This review critically examines recent advances in enhancing the stability of spinel Co_3_O_4_‐based catalysts for acidic OER. The fundamental reaction mechanisms of acidic OER are first analyzed to illustrate how different catalyst design strategies can be used to improve their stability. Next, five key catalyst design strategies reported in recent studies are summarized: 1) constructing protective surface layers, 2) modulating reaction pathways, 3) controlling cobalt redox dynamics, 4) tuning cobalt‐oxygen covalency, and 5) stabilizing lattice oxygen. Further, recent research progress in understanding the structure‐activity‐stability relationship of spinel Co_3_O_4_‐based catalysts is summarized, with a focus on identifying their catalytically active sites, tracking surface reconstruction, and elucidating degradation mechanisms. This review ends with a discussion of future research directions for addressing key challenges in realizing durable, high‐performance Co_3_O_4_‐based catalysts for acidic OER applications.

## Graphical abstract

This review systematically summarizes recent advances in enhancing the stability of spinel Co_3_O_4_‐based catalysts for the oxygen evolution reaction (OER) in acidic electrolytes. It discusses rational design strategies for obtaining durable Co_3_O_4_‐based OER catalysts, which can be applied in sustainable energy conversion and storage technologies, including water electrolysis, CO_2_ and N_2_ reduction, reversible fuel cells, and rechargeable metal‐air batteries.

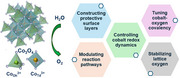

## 1 Introduction

The anodic oxygen evolution reaction (OER) is critical in water, CO_2_, and N_2_ electrolyzers, as well as reversible fuel cells and rechargeable metal‐metal batteries, for renewable electricity storage and sustainable fuel and chemical production. In particular, water electrolyzers powered by renewable electricity are promising for the sustainable production of hydrogen (H_2_).^[^ ^1^ ^]^ Proton exchange membrane (PEM) water electrolyzers operating in acidic electrolytes offer unique advantages over commonly used alkaline water electrolyzers, including lower ohmic losses, which enable higher current densities, produce H_2_ with higher purity, facilitate more compact electrolyzer designs, and allow for rapid electrolyzer responses.^[^ ^2^ ^]^ The anodic OER is a kinetically sluggish reaction involving four‐electron transfers with high overpotentials, which requires significant energy inputs.^[^ ^3^ ^]^ Noble metal oxide‐based catalysts, such as iridium oxides (IrO_x_) and ruthenium oxides (RuO_x_), have high catalytic activity and stability for acidic OER.^[^ ^4^ ^]^ However, their high cost has been a critical limiting factor for the large‐scale adoption of PEM water electrolyzers.^[^ ^5^ ^]^ Recent research efforts have focused on developing OER catalysts based on earth‐abundant materials.

**Figure 1:**
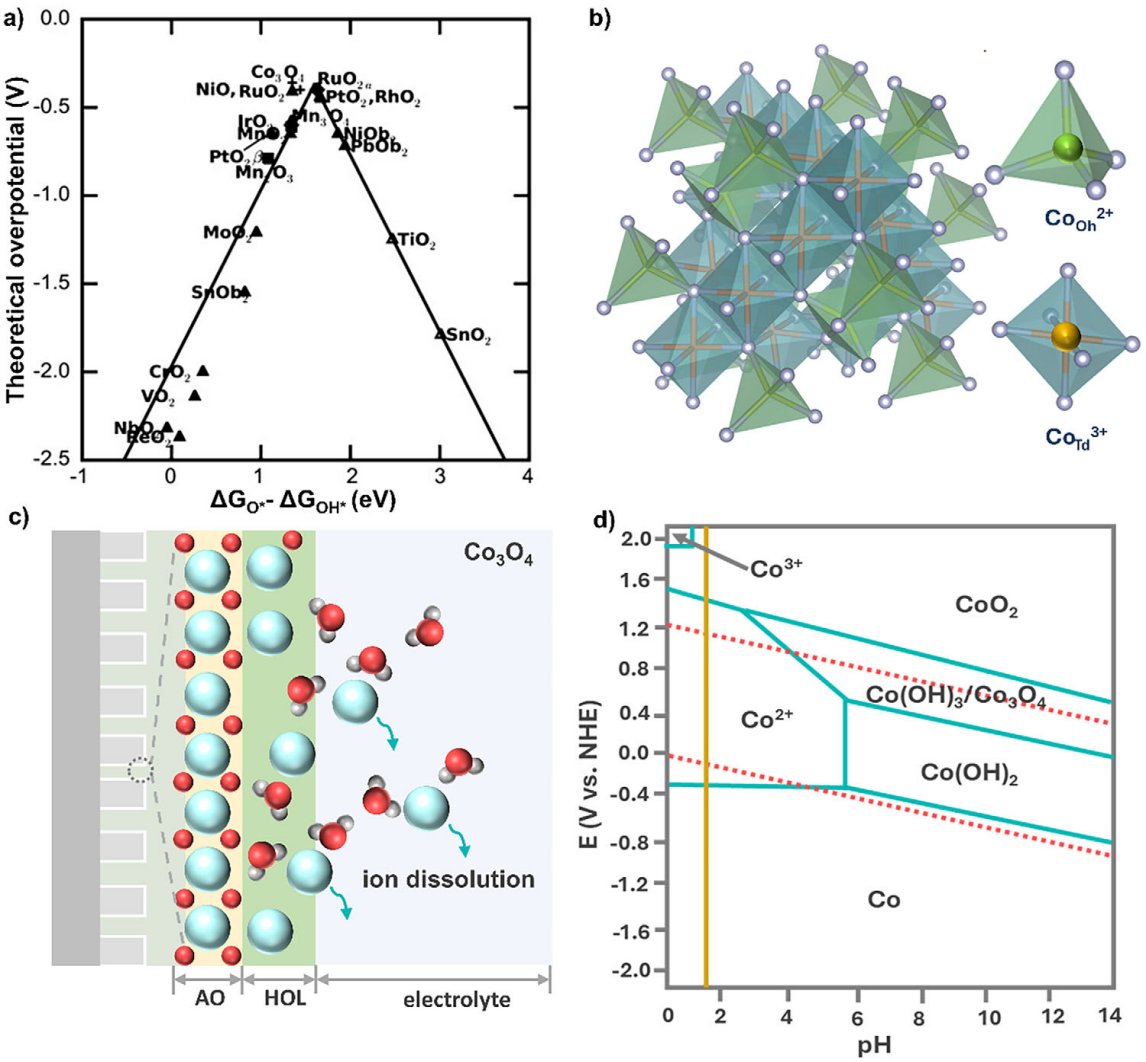
a) The theoretical catalytic activity of different metal oxide‐based OER catalysts. The negative values of OER overpotential are plotted versus ΔG_O*_−ΔG_HO*_, relative to the adsorption energies of OER intermediates on metal oxide surfaces. Reproduced with permission from ref. [17] Copyright 2011, Wiley. b) Atomic structure of spinel Co_3_O_4_ with octahedron (Oh) CoO_6_ and tetrahedron (Td) CoO_4_ units. c) Schematic illustration of the dissolution of spinel Co_3_O_4_ in acidic electrolytes. AO, anhydrous oxide; HOL, hydrous oxide layer. d) Simplified Pourbaix diagram of Co in aqueous solution with different pH. Green lines represent the borders of thermodynamic stability of various Co species. Dissolved species are considered to have an activity of 1. Red dashed lines mark the region of water stability, the lower line corresponding to the hydrogen evolution reaction and the upper line to OER. The vertical magenta line is drawn at pH = 1.6 as a guide to the eye. Reproduced and adapted with permission from ref.[18] Copyright 2014, ACS.

Transition metal oxide‐based catalysts have demonstrated excellent catalytic activity and stability for OER in alkaline electrolytes.^[^ ^6^ ^]^ Recent studies have shown that catalysts based on manganese oxides,^[^ ^7^ ^]^ cobalt oxides,^[^ ^8^ ^]^ iron oxides,^[^ ^9^ ^]^ and several multi‐metal oxides,^[^ ^10^ ^]^ also have high catalytic activities in acidic electrolytes. In particular, spinel Co_3_O_4_‐based catalysts exhibit moderate adsorption energies for OER intermediates, with theoretical catalytic activities comparable to those of RuO_2_ catalysts (**Figure** **1**a).^[^ ^11^ ^]^ Despite their competitive catalytic activity, spinel Co_3_O_4_‐based catalysts suffer from poor stability in acidic electrolytes. At potentials exceeding 1.47 V_RHE_ (vs reversible hydrogen electrode), even at low current densities (e.g., 0.1 mA cm^−2^), they would undergo severe dissolution of Co species, where CoO_2_, formed as an intermediate during OER, decomposes into soluble CoO accompanied by the release of oxygen.^[^ ^11^, ^12^ ^]^ The dissolution of spinel Co_3_O_4_ degrades catalyst activity and reduces their operational lifespan in acidic electrolytes.

Spinel Co_3_O_4_ consists of two types of Co sites: tetrahedral sites coordinated by four oxygen (O) atoms and octahedral sites coordinated by six O atoms (Figure 1b).^[^ ^13^ ^]^ In its standard spinel configuration, divalent Co^2+^ occupies the eight tetrahedral sites, while trivalent Co^3+^ resides in the 16 octahedral sites. The open lattice structure, combined with the presence of multiple accessible oxidation states, contributes to spinel Co_3_O_4_’s intrinsic OER activity but also makes it particularly vulnerable in acidic electrolytes. The high concentration of protons in acidic electrolytes exacerbates catalyst degradation through multiple pathways (Figure 1c). Proton‐induced ligand exchange weakens the Co─O lattice bonds, facilitating the leaching of Co ions from the surface lattice. This effect is particularly pronounced for tetrahedral Co^2+^, which is less shielded by the Co oxide matrix and thus more prone to dissolution, ultimately leading to the collapse of the entire spinel structure.^[^ ^14^ ^]^ Moreover, the elevated oxidative potentials required for OER in acidic conditions promote the formation of unstable high‐valent Co species (e.g., Co^3+^, Co^4+^), which are highly soluble. The degradation processes are further intensified by local pH fluctuations and ion concentration gradients near electrode interfaces, particularly under high current densities. In contrast, the degradation of Co_3_O_4_ in neutral and alkaline electrolytes is much slower. In alkaline electrolytes (e.g., pH > 12), Co_3_O_4_ tends to convert into more stable and catalytically active phases such as Co(OH)_2_ or CoOOH.^[^ ^13^, ^15^ ^]^ The reduced proton concentration in electrolytes diminishes the extent of proton‐driven lattice disruption. At the same time, any dissolved Co ions are more likely to re‐precipitate as stable hydroxides rather than diffuse away. Additionally, the formation of passivating surface layers in alkaline electrolytes may act as a catalyst surface protective barrier, further limiting Co dissolution and enhancing catalyst long‐term durability.

Moreover, the electrochemical stability of spinel Co_3_O_4_ is also pH dependent, with significantly accelerated degradation in acidic electrolytes. The Pourbaix diagram indicates the thermodynamic stability region of Co_3_O_4_ under various potential and pH conditions (Figure 1d).^[^ ^16^ ^]^ Beyond the stable region, Co_3_O_4_ can be oxidized into Co(OH)_3_, Co^3+^, and CoOH^2+^. Notably, the structural integrity of Co_3_O_4_ deteriorates rapidly when the applied potential exceeds 1.9 V_RHE_ at a pH of 0. Under anodic conditions, both Co^2+^ and Co^3+^ can be further oxidized to form soluble Co^3+^ and Co^4+^ species or transient high‐valent intermediates (e.g., CoO_2_), which dissolve readily in acidic electrolytes.^[^ ^12^ ^]^

The inherent instability of Co_3_O_4_ in acidic electrolytes poses a critical issue for spinel Co_3_O_4_‐based catalysts. Overcoming this challenge is imperative for realizing the deployment of these catalysts in PEM water electrolysis and other technologies that require robust stability in acidic electrolytes. Significant research efforts have been devoted to this task, which has been rarely reviewed to date. Herein, this review article systematically analyzes recent research advances in mitigating the instability of spinel Co_3_O_4_‐based OER catalysts. We begin by examining three different OER mechanisms on metal oxide‐based catalysts. Then, various strategies to improve spinel Co_3_O_4_’s stability in recent studies are discussed, including 1) constructing protective surface layers, 2) modulating reaction pathways, 3) controlling cobalt redox dynamics, 4) tuning cobalt‐oxygen covalency, and 5) stabilizing lattice oxygen. Next, the critical roles of using *operando* characterization techniques in identifying catalytically active sites, tracking catalyst surface reconstruction, and uncovering catalyst degradation pathways during OER are highlighted. Finally, we summarize existing challenges and propose potential research directions to develop active and stable spinel Co_3_O_4_‐based OER catalysts for PEM water electrolysis.

## 2 OER Mechanisms

A clear understanding of OER mechanisms is essential to designing high‐performance OER catalysts. To date, three types of mechanisms have been proposed for metal oxide‐based OER catalysts, each closely associated with the catalyst's geometric and electronic structures.

### 2.1 Adsorbate Evolution Mechanism (AEM)

**Figure 2:**
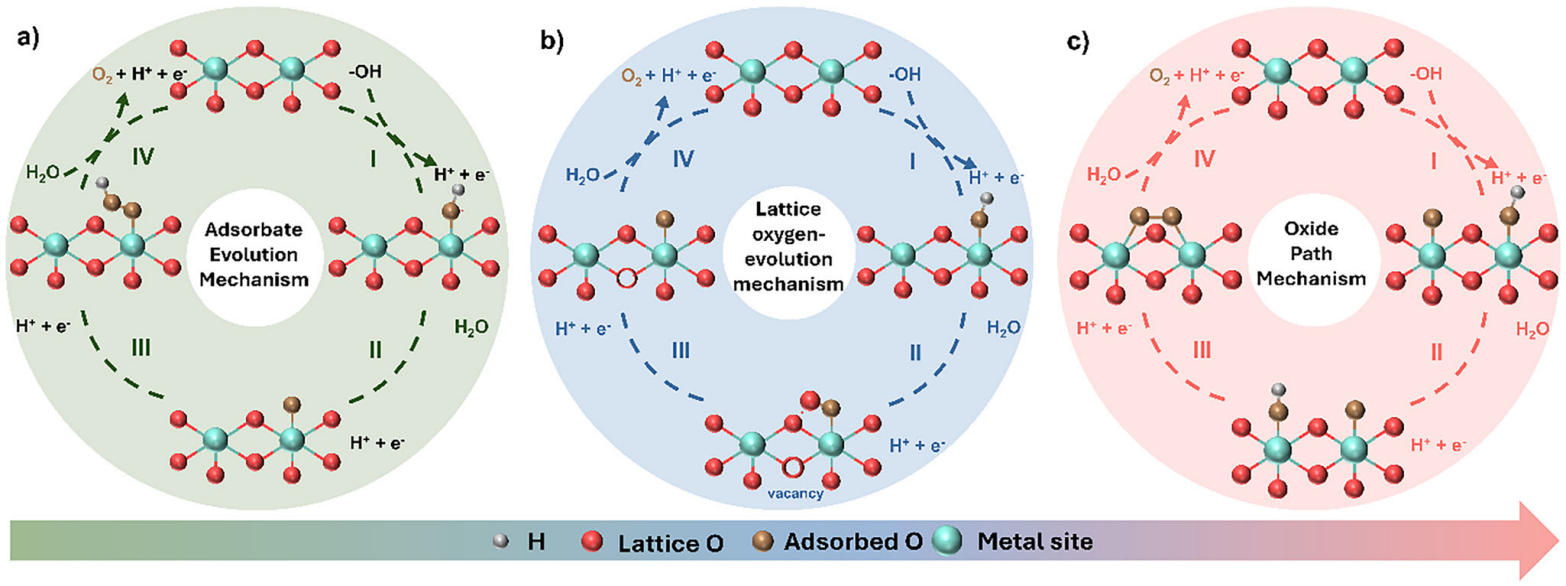
Schematic illustration of three OER mechanisms: a) adsorbate evolution mechanism (AEM), b) lattice oxygen‐evolution mechanism (LOM), and c) oxide path mechanism (OPM).

AEM proposes that OER proceeds in four reaction steps, with each step involving a proton‐coupled electron transfer over a metal active site on catalyst surfaces (**Figure** **2**a). Oxygenated intermediates, such as OH^−^ or H_2_O, first adsorb on an active site, forming OH*. Next, the deprotonation of OH* generates O*. Subsequently, O* undergoes nucleophilic attack by OH^−^ or H_2_O to form OOH*, which ultimately evolves into an O_2_ molecule and detaches from the active sites. Koper et al. proposed a general scaling relationship between the binding energies of OH* and OOH* on a metal active site with a constant energy difference of 3.2 ± 0.2 eV (i.e., ∆G_OOH*_‐∆G_OH*_).^[^ ^19^ ^]^ This scaling relationship suggests that either ∆G_OH*_ or ∆G_OOH*_ can act as the rate‐determining step (RDS) in OER. Since O* is in the middle of this scaling relationship, the value of Δ*G*_O* –_ Δ*G*_OH*_ has been commonly used to predict an OER catalyst's catalytic activity. Montoya et al. illustrated this correlation between Δ*G*_O* –_ Δ*G*_OH*_ and catalytic activity of metal oxide‐based OER electrocatalysts in a volcano plot, suggesting a minimum theoretical overpotential of 370 mV for OER via AEM.^[^ ^20^ ^]^ Thus, the catalytic activity of catalysts is constrained according to AEM. Breaking the linear scaling relationship is necessary to achieve catalytic activity exceeding this theoretical limit.

### 2.2 Lattice Oxygen‐Evolution Mechanism (LOM)

The initial reaction steps proposed in LOM involve the generation of O* and OH* intermediates that are analogous to those in AEM (Figure 2b). However, the surface O* intermediate interacts with lattice oxygen in metal oxide catalysts, leading to direct O─O bond formation in LOM. The resulting surface vacancy is replenished by an H_2_O molecule, which generates OH* species and releases a proton via a one‐electron oxidation process.^[^ ^21^ ^]^ OER via LOM has an advantage by circumventing the overpotential limit suggested by AEM because the formation of OOH* is bypassed.^[^ ^22^ ^]^ Besides, catalytically active sites in LOM are not limited to metal sites. Lattice oxygen in catalysts also participates in OER and is an additional active site. However, metal oxides have stability issues in LOM due to bulk oxygen diffusion and structural evolution triggered by the continuous formation of oxygen vacancies and the dissolution of cations during lattice oxygen redox reactions. This phenomenon has been attributed to the higher dissolution rate observed in electrochemically synthesized (defect‐rich) RuO_2_ compared to its thermally synthesized counterpart.^[^ ^23^ ^]^ In other words, the enhancement of OER activity via LOM in transition metal oxide‐based catalysts often comes at the expense of their stability. An ongoing debate exists regarding whether lattice oxygen in transition metal oxide‐based catalysts should be fully suppressed, partially suppressed, or activated to enhance their catalytic activity and stability for OER.

### 2.3 Oxide Path Mechanism (OPM)

OER proceeds via OPM may overcome the theoretical limit suggested by AEM and bypass the stability issue in LOM.^[^ ^24^ ^]^ OPM leverages two metal sites with an optimal atomic distance to facilitate direct O─O radical coupling, whereby only O* and OH* are involved as OER intermediates, leading to O_2_ formation and release without the involvement of additional protons or electrons to form OOH* (Figure 2c). Therefore, OER, according to OPM, could efficiently break the linear scaling relationship in AEM, resulting in lower overpotential and higher overall energy efficiency. In addition, lattice oxygen remains intact in OPM, and OER occurs solely through surface‐active sites. This eliminates the need for lattice oxygen vacancy formation, alleviating catalysts’ structural collapse and ensuring long‐term stability. However, OPM imposes more stringent requirements on the geometric and electronic configuration of active sites in OER catalysts. To drive OER via the reaction pathway suggested by OPM requires precise control over the atomic distance between two adjacent active sites and their electronic properties to promote effective O‐O radical coupling.

## 3 Strategies for Improving the Stability of Spinel Co_3_O_4_‐Based Catalysts

Significant research efforts have been devoted to enhancing the stability of spinel Co_3_O_4_‐based OER catalysts in acidic electrolytes. These efforts aim to optimize the coordination environment of Co ions and regulate their geometric structures. In the following sections, we summarize studies based on five different approaches: 1) constructing protective surface layers, 2) modulating reaction pathways, 3) controlling Co redox dynamics, 4) tuning Co─O covalency, and 5) stabilizing lattice O.

### 3.1 Constructing Protective Surface Layers

**Figure 3:**
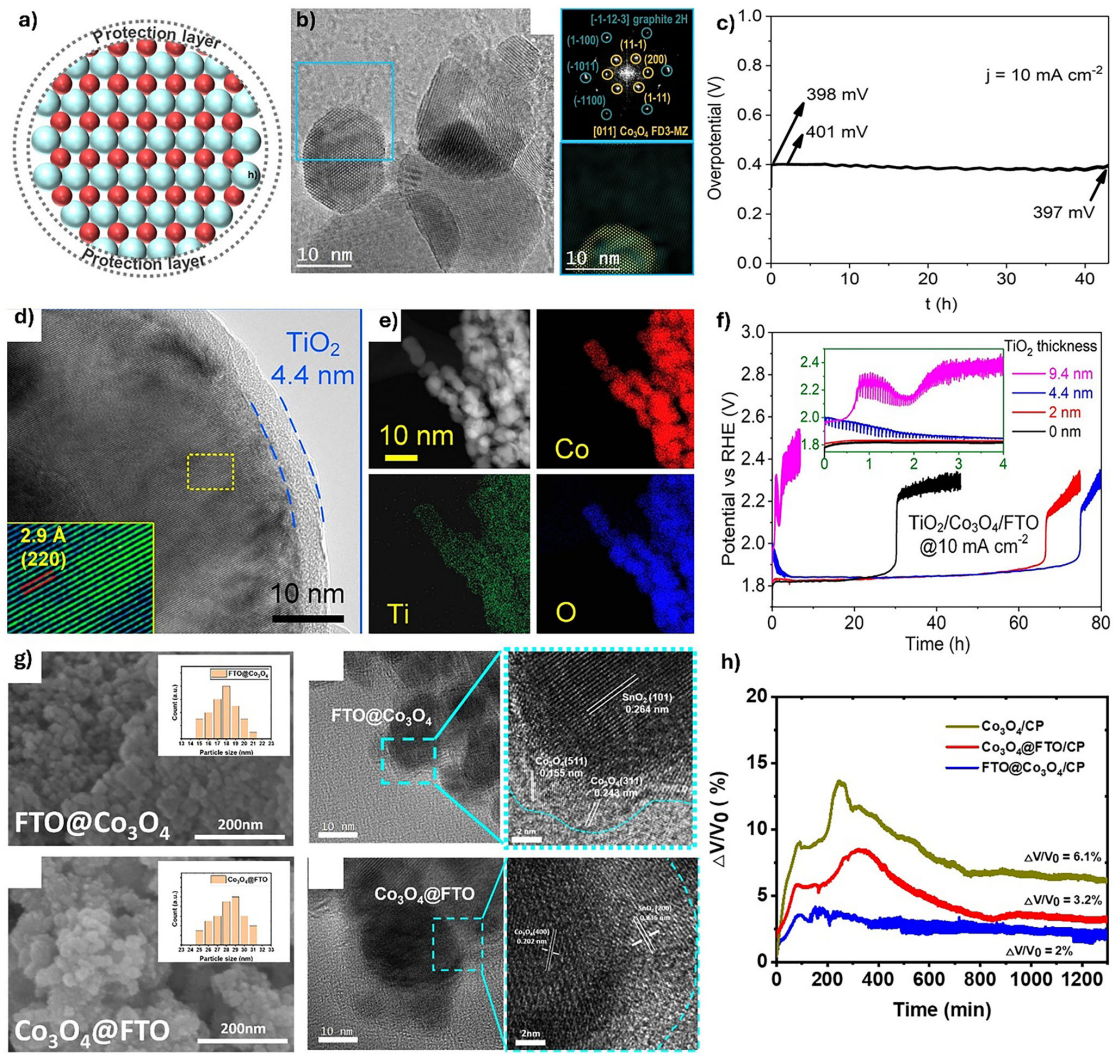
a) Schematic illustration of protective surface layer construction strategies for enhancing the stability of Co_3_O_4_‐based catalyst. b) TEM image of Co_3_O_4_@C/GPO. c) Stability of Co_3_O_4_@C/GPO over 40 h. Reproduced with permission from ref. [26] Copyright 2022, Springer Nature. d) TEM and e) elemental mappings of TiO_2_/coated Co_3_O_4_. f) Stability test of TiO_2_/Co_3_O_4_ with different TiO_2_ coating thicknesses. Reproduced with permission from ref.^[25b]^ Copyright 2022, ACS. g) SEM and TEM images and i) Stability test of FTO@Co_3_O_4_ and Co_3_O_4_@FTO. Reproduced with permission from ref.[25c] Copyright 2023, RSC.

Spinel Co_3_O_4_ generally suffers from severe dissolution, structural collapse, and loss of active sites as an OER catalyst in acidic electrolytes, significantly impacting its stability even at low current densities. Surface protective layers made of different materials, such as carbon materials or acid‐resistant metal oxides (e.g., TiO_2_), can act as physical and/or chemical barriers between Co_3_O_4_ and acidic electrolytes, thereby minimizing the direct exposure of catalytically active sites to harsh acidic environments (**Figure** **3**a).^[^ ^25^ ^]^

For example, Yu et al. synthesized graphite and paraffin oil‐coated Co_3_O_4_ nanoparticles (Co_3_O_4_@C/GPO), showing robust stability for acidic OER over 40 h (Figure 3b,c).^[^ ^26^ ^]^ The hydrophobic surface of Co_3_O_4_@C/GPO effectively suppresses Co leaching, thereby maintaining its catalytically active sites and enabling stable catalytic performance. However, carbon material‐based surface protective layers experience corrosion at potentials exceeding 1.2 V_RHE_, even under room temperature conditions, particularly at high current densities.^[^ ^27^ ^]^ Alternatively, Tran‐Phu et al. coated a TiO_2_ protective layer over Co_3_O_4_ particles with adjustable thicknesses (TiO_2_/Co_3_O_4_), demonstrating 80 h stability for acidic OER, 3 times longer compared to pristine Co_3_O_4_ without coating (Figure 3d–f).^[^ ^25b^ ^]^ However, the enhanced stability of TiO_2_/Co_3_O_4_ via the surface protective TiO_2_ layer comes at the cost of reduced catalytic activity due to higher charge‐transfer resistance caused by the nonconductive TiO_2_ layer. Henceforth, other protective layers with anti‐corrosion and higher electroconductivity have been explored. For example, Yeh et al. mixed fluorine‐doped tin oxide (FTO) with Co_3_O_4_ (Figure 3g).^[^ ^25c^ ^]^ Co_3_O_4_‐deposited on FTO (FTO@Co_3_O_4_) exhibits a much higher catalytic activity and lower decay rate than FTO‐coated Co_3_O_4_ (Co_3_O_4_@FTO) in acidic electrolytes (Figure 3h). The improved stability of FTO@Co_3_O_4_ was attributed to the dispersion of Co_3_O_4_ on FTO, which exposed catalytically active sites while FTO effectively suppressed Co leaching. When utilized as the anode in a PEM water electrolyzer, achieving a current density of 0.205 A cm^−2^ at 2 V under ambient conditions, and demonstrated stability, exhibiting only a 5.8% increase in cell voltage after 21.5 h at 10 mA cm^−2^. Nevertheless, its performance under high current densities and elevated temperatures has yet to be investigated. Accordingly, constructing surface protective layers on Co_3_O_4_ can mitigate its leaching in acidic electrolytes. However, such protective layers may block catalytically active sites. In this regard, selecting appropriate coating materials and optimizing their thickness is crucial to obtaining optimized outcomes.

### 3.2 Modulating Reaction Pathways

**Figure 4:**
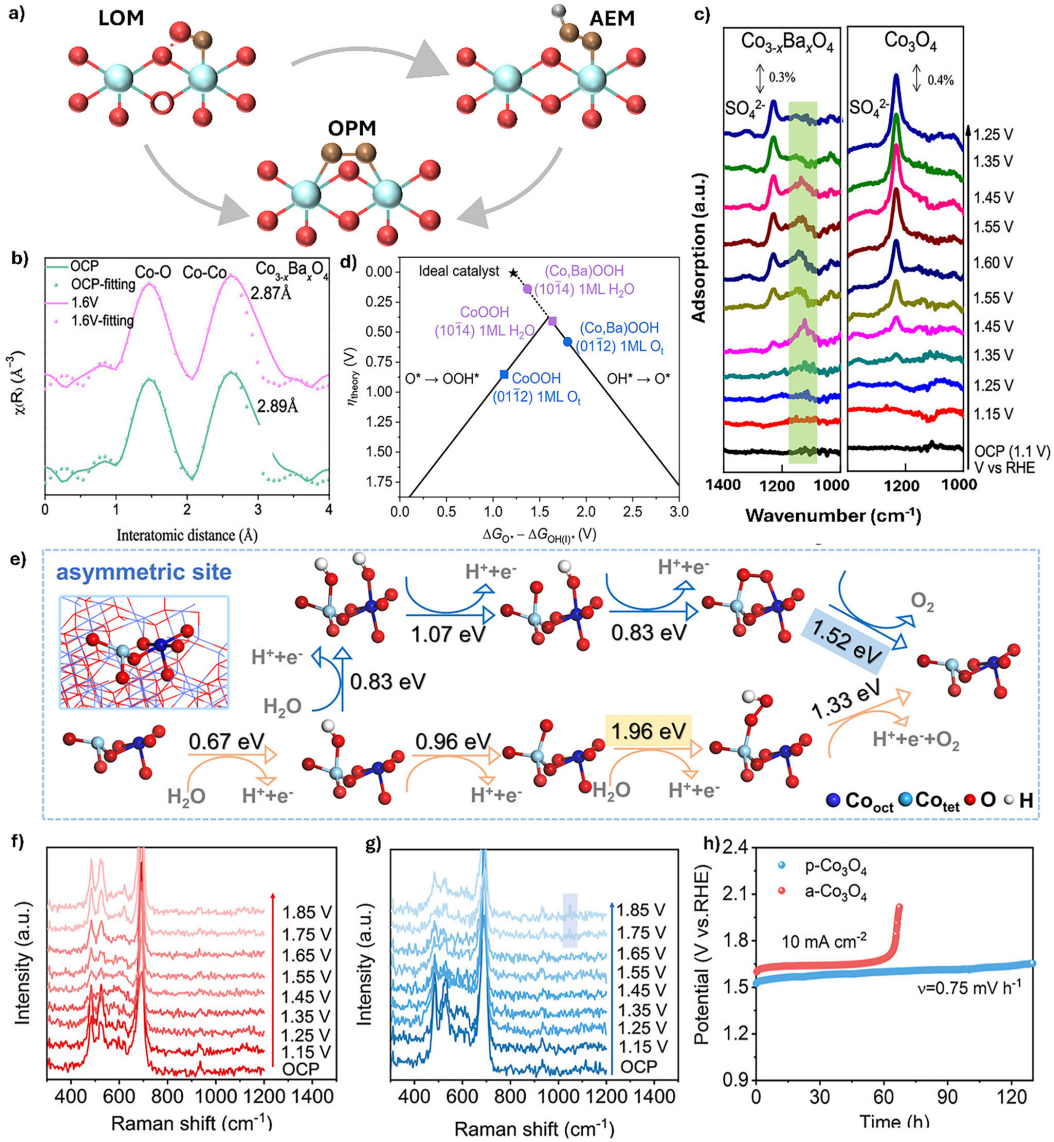
a) Schematic illustration of the modulation of reaction pathways for enhancing the stability of Co_3_O_4_‐based catalyst. b) In situ extended XAS of the Co *K*‐edge from Co_3–_ *_x_* Ba*_x_* O_4_ at OCP and 1.6 V versus RHE. c) In situ FTIR spectra recorded in the potential range of OCP to 1.6 V versus RHE for Co_3–_ *_x_* Ba*_x_* O_4_ and Co_3_O_4_. d) Surface free energy for different slabs of Ba‐doped Co_3_O_4_ and CoOOH as a function of applied potential at pH = 0 (up), and OER volcano plot showing the predicted theoretical overpotential versus the free energy difference between the formation of O* and OH* (down). Reprinted with permission from ref.[28] Copyright 2023, ACS. e) Free energy of reaction steps along the AEM (orange) and OPM (blue) pathways on Co_oct_‐Co_tet_ sites. In situ Raman of f) a‐Co_3_O_4_ and g) p‐Co_3_O_4_. h) Chronopotentiometry test of p‐Co_3_O_4_ and references in 0.1 M HClO_4_. Reprinted with permission from ref.[29b] Copyright 2024, ACS.

As discussed in Section 2.3, OER via the OPM pathway, which involves direct O─O bond formation without generating oxygen vacancies or additional reaction intermediates (OOH*), can significantly enhance both the activity and stability of catalysts. Thus, modulating the reaction pathway of spinel Co_3_O_4_‐based catalysts toward the OPM route offers a promising strategy to improve their performance, particularly under acidic conditions. This modulation can be achieved by tuning the local coordination environment of cobalt within the spinel structure, thus stabilizing metal cations or oxygen anions for stable operation (**Figure** **4**a). For example, Wang et al. doped Ba cations into Co_3_O_4_ (Co_3‐x_Ba_x_O_4_), which efficiently triggered the OPM pathway for acidic OER.^[^ ^28^ ^]^ The resulting Co_3‐x_Ba_x_O_4_ exhibits an overpotential of 278 mV at a current density of 10 mA cm^−2^ in 0.5 m H_2_SO_4_, demonstrating excellent stability over 110 h. In situ X‐ray absorption spectroscopy (XAS) results show that the Co_3–x_Ba_x_O_4_ catalyst shows a shorter Co─Co distance under applied potential compared to pristine Co_3_O_4_ (Figure 4b). Besides, in situ synchrotron fourier transform infrared spectroscopy (FTIR) shows a distinctive absorption peak at 1122 cm^−1^ at 1.45 V_RHE_, suggesting the generation of an O─O bond, consistent with oxygen bridges between adjacent Co metal sites in the OPM pathway (Figure 4c). These experimental observations are further supported by theoretical calculations, which show that surface‐adsorbed Ba atoms reduce the surface free energy of CoOOH and facilitate both deprotonation and O─O bond formation (Figure 4d).

Beyond heteroatom doping, defect engineering also offers an effective means to tailor reaction pathways.^[^ ^29^ ^]^ Cui et al. conducted a comprehensive theoretical analysis of OER on pristine Co_3_O_4_. They found that the OPM, proceeding through the Co_oct_–Co_tet_ dual site, exhibits a lower RDS free energy barrier compared to the conventional AEM (Figure 4e).^[^ ^29b^ ^]^ However, the O‐─‐O coupling efficiency is hindered by the relatively large interatomic distance between the two Co atoms. With the introduction of O vacancies, the resulting asymmetric active sites composed of one O‐deficient Co_oct_ and one intact Co_oct_ effectively reduce the Co─Co distance and enhance the feasibility of direct O─O bond coupling via the OPM pathway. Theoretical insights were corroborated by operando Raman spectroscopy performed on plasma‐treated Co_3_O_4_ (p‐Co_3_O_4_), which revealed a distinctive vibrational peak at 1050 cm^−1^ at 1.75 V versus RHE, assigned to the direct O─O coupling via the OPM pathway (Figures 4f,g). As a result, the p‐Co_3_O_4_ catalyst demonstrates an overpotential of 287 mV at 10 mA cm^−2^ and a low degradation rate of 0.75 mV h^−1^ in 0.5 m H_2_SO_4_ (Figure 4h). These results demonstrate the role of lattice defects in promoting favorable active site configurations and shifting the OER mechanism toward direct O–O coupling on defective Co_3_O_4_ surfaces.

### 3.3 Controlling Co Redox Dynamics

Spinel Co_3_O_4_‐based catalysts usually undergo surface reconstruction during OER, involving stepwise redox reactions of Co species (Co^2+^Co^3+^ → Co^3+^Co^3+^ → Co^3+^Co^4+^ → Co^4+^Co^4+^).^[^ ^30^ ^]^ Several in situ/operando spectroscopic studies have shown the phase transformation from Co^3+^OOH to CoO_x_ (likely Co^4+^ in CoO_2_ with the structure of Co^4+^ = O) under OER conditions.^[^ ^30^, ^31^ ^]^ Co(IV)═O and Co(III)OH may serve as catalytically active sites for OER with different reaction kinetics.^[^ ^30d^ ^]^ However, excessive oxidation to Co^4+^‐rich phases can lead to irreversible structural degradation, such as lattice oxygen loss or phase segregation, compromising long‐term stability.

**Figure 5:**
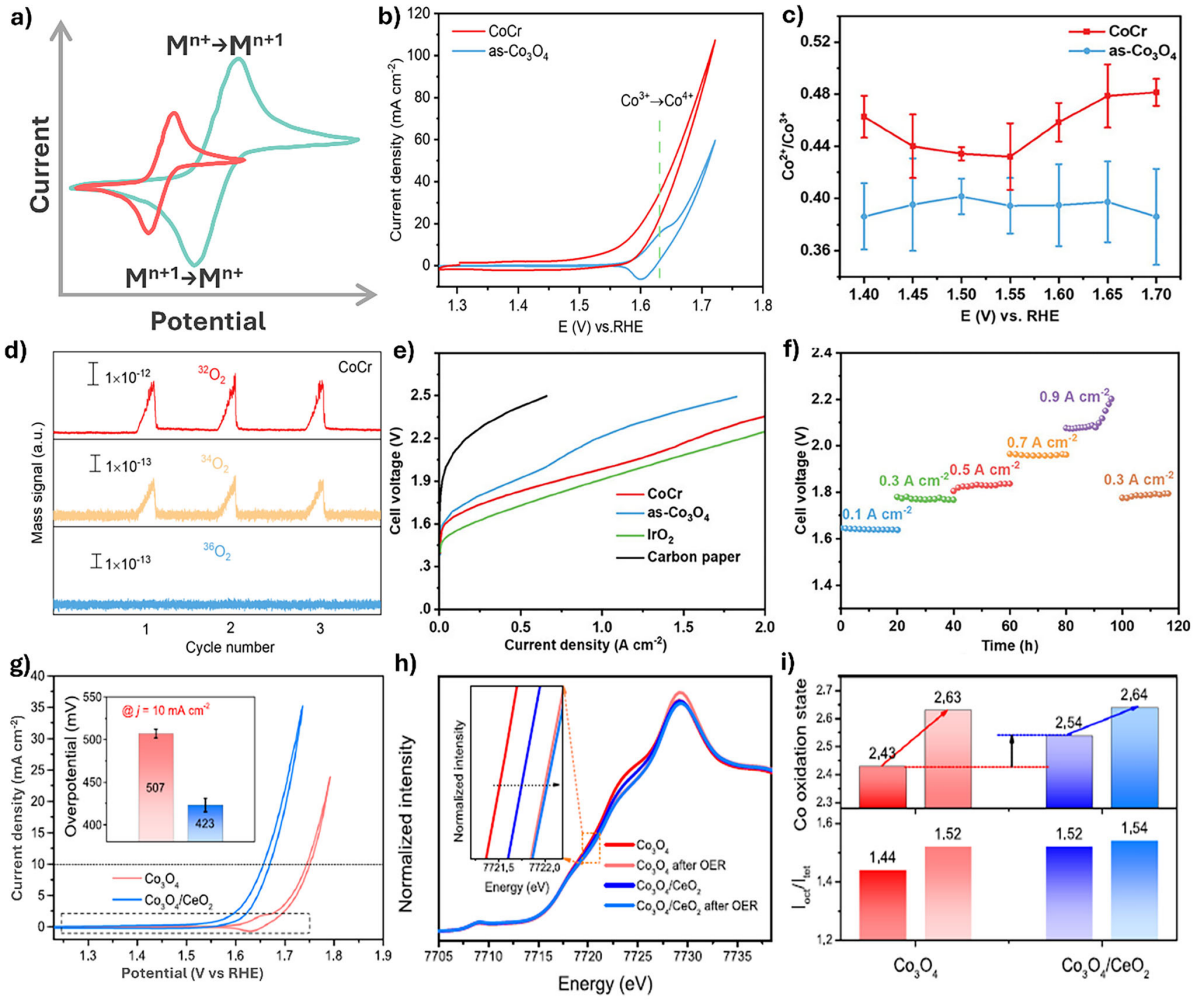
a) Schematic illustration of the regulation of redox behavior for enhancing the stability of Co_3_O_4_‐based catalyst. b) CV curves of CoCr and as‐Co_3_O_4_ catalysts in 0.5 m H_2_SO_4_. The CoCr catalyst shows no obvious Co^3+^/^4+^ redox feature. c) Co^2+^/Co^3+^ ratio plotted against the applied potential, and d) DEMS signals of O_2_ products for CoCr and as‐Co_3_O_4_ catalysts. e) Current‐voltage polarizations of CoCr and references in PEWMEs, and f) Chronopotentiometry curves of the PEMWE using CoCr at different current densities. Reprinted with permission from ref.[32] Copyright 2024, Wiley. g) CV curves of Co_3_O_4_ and Co_3_O_4_/CeO_2_ in 0.5 M H_2_SO_4_ (inset: required overpotential to achieve 10 mA cm^−2^). h) Co K‐edge XAS spectra and i) Average Co oxidation states for Co_3_O_4_ and Co_3_O_4_/CeO_2_ before and after OER. Reproduced with permission from ref.[30a] Copyright 2021, Springer Nature.

To this end, tuning the redox behavior of spinel Co_3_O_4_ may optimize the population of active sites and simultaneously mitigate stability‐limiting processes, such as over‐oxidation, Co^3+^ dissolution, and irreversible phase transformations (**Figure** **5**a). For example, Yan et al. found that the incorporation of Cr increases the oxidation state of Co and enhances the covalency and flexibility of Co─O bonds, thereby accelerating electron transfer and the conversion of Co^3+^ to the active high oxidation state Co^4+^, which facilitates OER activity (Figure 5b).^[^ ^32^ ^]^ Notably, the operando Raman spectroscopy studies show that the Cr dopants accelerate the pre‐oxidation process to generate active Co^4+^ species at a lower potential than that in the pristine Co_3_O_4_, where the quick Co^3+/4+^ redox efficiently breaks the activity/stability trade‐off for acidic OER via the AEM pathway, as evidenced by their operando differential electrochemical mass spectroscopy (DEMS) results (Figure 5c,d). The CoCr catalyst achieved 1.5 A cm^−2^ at 2.17 V and exhibited notable durability over 100 h at 0.50 A cm^−2^ and 500 h at 0.1 A cm^−2^, demonstrating promising potential for future industrial applications (Figure 5e,f). Additionally, Wang et al. doped fluorine (F) into Co_3_O_4_, creating geometrically reconstructed Co sites, F─Co─O.^[^ ^33^ ^]^ F induces an electron‐dominated sharing effect that modulates the redox behaviors of Co in spinel Co_3_O_4_. The resulting Co_3_O_4‐x_F_x_ catalyst demonstrates stable operation for 120 h at 100 mA cm^−2^ for acidic OER. Ex situ Co K‐edge XAS results show that the average Co oxidation state in Co_3_O_4‐x_F_x_ increases progressively with the applied potential while remaining lower than that of the initial Co_3_O_4_, suggesting that the F dopants effectively modulate the local microenvironment of the F─Co─O active sites for improving both activity and crystal structural stability in acids.

The redox behavior of Co_3_O_4_ can also be adjusted by constructing heterogeneous structures with other oxides to regulate its electronic structure.^[^ ^34^ ^]^ For example, Huang et al. incorporated nanocrystalline CeO_2_ into a Co_3_O_4_/CeO_2_ composite.^[^ ^35^ ^]^ The resulting Co_3_O_4_/CeO_2_ exhibits a lower overpotential of 423 mV at a current density of 10 mA cm^−2^ compared to pure Co_3_O_4_ (507 mV) (Figure 5g). Notably, Co_3_O_4_/CeO_2_ exhibited a 2.5‐fold reduction in Co dissolution compared to pure Co_3_O_4_ under open‐circuit conditions. Physical characterizations reveal that nanocrystalline CeO_2_ alters the electronic structure of Co_3_O_4_, creating a more favorable local bonding environment, which facilitates the oxidation of surface Co^3+^ species into Co^4+^ species while reducing charge accumulation during OER. Notably, the valence state of Co atoms in Co_3_O_4_/CeO_2_ and the intensity ratio (I_oct_/I_tet_, oct: octahedral Co site; tet: tetrahedral Co site) ratio shows minor changes after OER (Figure 5h,i).

### 3.4 Tuning Co─O Covalency

**Figure 6:**
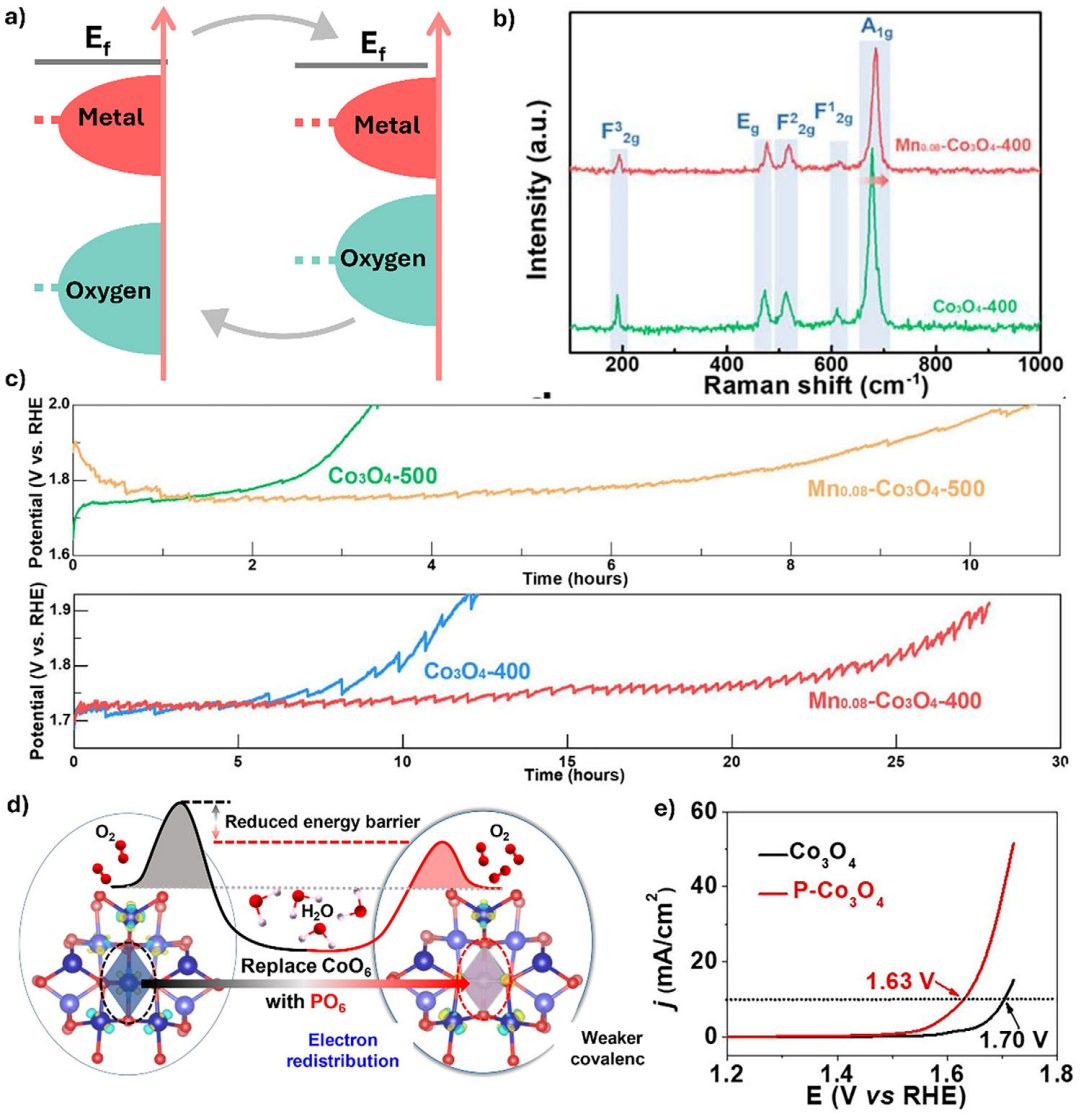
a) Schematic illustration of the regulation of metal‐oxygen covalency for enhancing the stability of Co_3_O_4_‐based catalyst. b) Raman spectra of Mn_0.08_‐Co_3_O_4_‐400 and Co_3_O_4_‐400. c) Chronopotentiometry curves of Mn_0.08_‐Co_3_O_4_‐400 and referencs in 0.5 m H_2_SO_4_. Reprinted with permission from ref.[39] Copyright 2023, Elsevier. d) Schematic illustration of metal‐O covalency before and after introducing P dopants. e) Polarization curves of P‐Co_3_O_4_ and Co_3_O_4_ in 0.1 m HClO_4_. Reprinted with permission from ref.[40] Copyright 2023, Elsevier.

Metal–O covalency refers to the extent of covalent character in the bond between metal cations and oxygen anions within a compound or catalytic material.^[^ ^36^ ^]^ The strength of metal–O covalency affects the electronic structure of the catalyst, such as the density of states near the Fermi level (**Figure** **6**a).^[^ ^37^ ^]^ In general, catalytic materials with high metal‐O covalency increase the orbital overlap between metal d‐orbitals and O p‐orbitals, exerting a significant effect on OER performance by adjusting the adsorption energy of oxygenated intermediates, optimizing the electronic structure, and potentially stabilizing metal cations or oxygen anions.^[^ ^8^, ^38^ ^]^

To leverage these effects, several studies have tried to dope other metals/elements into Co_3_O_4_ to regulate metal‐O covalency and influence the stability of Co_3_O_4_ in OER. For example, Fan et al. reported a catalyst based on cavity‐rich manganese (Mn) doped Co_3_O_4_ spinel nanosheets (Mn_x_‐Co_3_O_4_) for acidic OER, which exhibited a 3 times longer lifetime at a high voltage of 1.8 V_RHE_ (Figure 6b,c).^[^ ^39^ ^]^ The improved stability was attributed to the formation of Mn─O─Co bonds in Mn_x_‐Co_3_O_4_, which enhances the Co─O covalency by increasing Co 3d–O 2p orbital hybridization. This enhanced covalency promotes electronic delocalization, stabilizing the Co oxidation state during redox cycling, thereby preserving structural integrity under acidic conditions. Nonetheless, it is essential to finely control the extent of covalency, as excessive Co─O interaction can lead to unfavorable lattice O participation and potential catalyst degradation.

Conversely, Shang et al. introduced phosphorus atoms into the spinel lattice of spinel Co_3_O_4_ (P‐Co_3_O_4_) to partially substitute octahedral Co^3+^ sites, achieving improved stability in acidic OER.^[^ ^40^ ^]^ This modification resulted in the formation of PO_6_ structural motifs and a redistribution of electron density around neighboring Co sites (Figure 6d,e). The local electronic perturbation effectively reduced Co─O covalency, which in turn suppressed the involvement of lattice O in the OER via the LOM. Instead, this lowered covalency favored the AEM, contributing to greater structural robustness and long‐term stability. Notably, decreasing Co─O covalency proves beneficial by preserving the crystallinity and integrity of the catalyst surface, particularly under acidic and high‐potential conditions where LOM‐induced instability is more pronounced.

These contrasting examples underscore the context‐dependent nature of Co─O covalency in determining OER activity and stability. Three interrelated factors may reconcile the seemingly contradictory findings. First, increased metal‐O covalency generally facilitates stronger bonding with O intermediates, lowering energy barriers for key OER steps and enhancing catalytic activity.^[^ ^41^ ^]^ It may also stabilize metal centers against demetallation during electrolysis for stable operation.^[^ ^42^ ^]^ However, overly strong covalency can hinder the desorption of intermediates, thus impeding the overall reaction kinetics. Second, excessively high metal‐oxygen covalency might switch the reaction pathway from AEM to LOM, potentially destabilizing the catalysts’ structure during OER.^[^ ^43^ ^]^ Third, intense hybridization between metal d‐orbitals and oxygen p‐orbitals may induce surface amorphization or lattice degradation, particularly under prolonged electrochemical operation.^[^ ^44^ ^]^ Therefore, rationally tuning the metal‐oxygen covalency is essential for designing Co_3_O_4_‐based catalysts that simultaneously achieve high activity and long‐term durability. Notably, a deeper mechanistic understanding should account for the specific structural and electronic effects induced by different dopants, such as metal and non‐metal dopants, which ultimately govern the reaction pathway and the catalyst's resistance to degradation.

### 3.5 Stabilizing Lattice O

**Figure 7:**
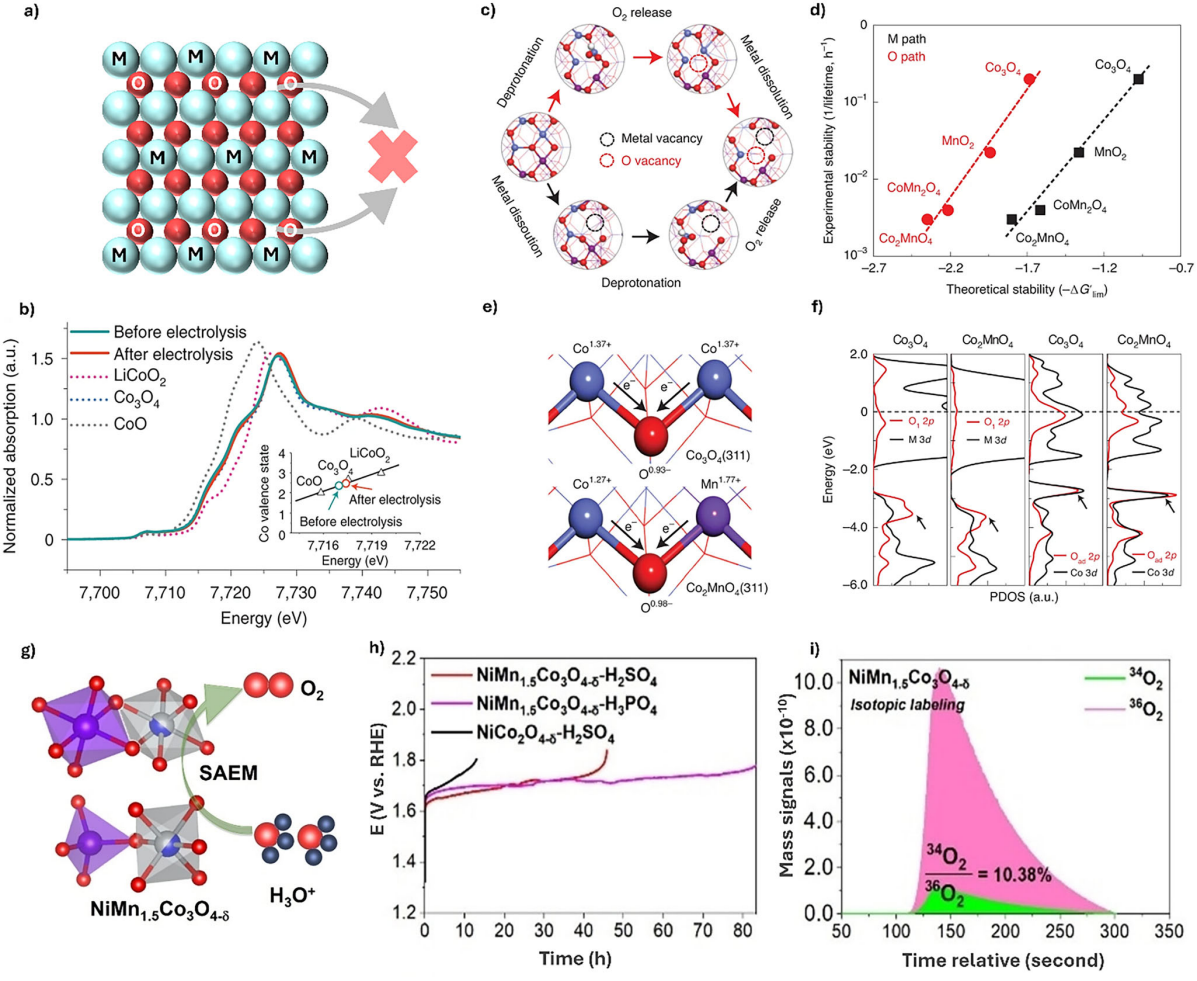
a) Schematic illustration of the stabilization of lattice oxygen for enhancing the stability of Co_3_O_4_‐based catalyst. b) Normalized Co K‐edge XANES spectra of Co_2_MnO_4_ before and after OER. c) Schematic illustration of Co_2_MnO_4_ dissolution mechanism in acids. d) The experimental dissolution rate at 100 mA cm^−2^ (1/lifetime, h^−1^) and the thermodynamic limiting barrier towards dissolution (–ΔG_lim_) obtained from theoretical calculations. e) The different oxidation states of Co_3_O_4_ and Co_2_MnO_4_ indicated by electron transfer via Bader charge calculations. The black arrows show the direction of electron transfers. f) PDOS results of lattice O 2p and connected metal 3d orbitals, and as adsorbed *O 2p with surface Co 3d orbitals. Reproduced with permission from ref.[47] Copyright 2022, Springer Nature. g) Schematic illustration of NiMn_1.5_Co_3_O_4‐δ_ structure. h) The long‐term stability of NiMn_1.5_Co_3_O_4‐δ_ and references. i) Isotopic labeling differential electrochemical mass spectrometry tests of NiCo_2_O_4‐δ_. Reproduced with permission from ref.[10b] Copyright 2024, Wiley.

The over‐oxidation of spinel Co_3_O_4_ during OER in acidic electrolytes is accompanied by the oxidation of lattice O, resulting in O vacancy formation. The generated oxygen vacancy would accelerate Co dissolution, leading to the structural collapse of Co_3_O_4_ catalysts.^[^ ^45^ ^]^ It is commonly accepted that the degradation of the spinel Co_3_O_4_ catalyst's catalytic activity in acidic OER is primarily caused by the loss of lattice O and Co leaching (**Figure** **7**a).^[^ ^46^ ^]^ Therefore, several studies have explored heteroatom doping engineering to stabilize lattice O.

For example, Xiao et al. demonstrated that the partial substitution of Co at octahedral sites (Co_oct_) with Mn^3+/4+^ enhances its stability and maintains its activity compared to that of pristine Co_3_O_4_.^[^ ^47^ ^]^ Co_2_MnO_4_ demonstrates stable operation over 1500 h at a high current density of 200 mA cm^−2^ for acidic OER. Physical characterizations and theoretical analysis results suggest that Mn substitution facilitated more electron transfer from Mn to O, forming a stronger Mn─O bond. The interaction between the lattice O 2p orbitals and Mn 3d orbitals in Co_2_MnO_4_ exhibits a more stable Mn─O bond compared to the Co─O bond in Co_3_O_4_, significantly improving stability and preventing the dissolution of lattice oxygen atoms (Figure 7b–f). Similarly, Zhao et al. introduced Mn dopants to decrease the concentration of oxygen vacancy and form Mn‐O structure adjacent octahedral sites in spinel NiCo_2_O_4‐δ_ (NiMn_1.5_Co_3_O_4‐δ_), delivering long‐term stability of 80 h in acidic electrolytes (Figure 7g,h).^[^ ^10b^ ^]^ The isotopic labeling experiment suggests the conventional AEM pathway on NiMn_1.5_Co_3_O_4‐δ_ for acidic OER (Figure 7i). Theoretical study results indicate that Mn dopants effectively increase the localized gap between O 2p band centers and the Fermi level, enhancing the oxygen vacancy formation energy of NiCo_2_O_4‐δ_ for stable OER operation. Also, a bimetallic‐doped Co‐based oxide–titanium diboride composite catalyst (Ce‐Mn‐Co_3_O_4_/TiB_2_) has been reported to exhibit both high activity and stability for acidic OER.^[^ ^48^ ^]^ The Ce‐dopants introduce surface O vacancies, which modulate the adsorption energies of reaction intermediates, thereby enhancing catalytic activity. Concurrently, the Mn‐dopants play a critical role in stabilizing lattice O and suppressing metal ion dissolution, contributing to improved durability. As a result, the Ce‐Mn‐Co_3_O_4_/TiB_2_ catalyst delivers current densities of 0.25 and 1 A cm^−2^ at 1.63 and 2.2 V_RHE_, respectively, and maintains stable performance at 0.25 A cm^−2^ for 25 h in a PEM water electrolyzer.

Collectively, these studies suggest that improving the O vacancy formation energy and reinforcing metal‐O─O bonds in spinel Co_3_O_4_ structures are key strategies for enhancing stability under acidic OER conditions. Increasing the vacancy formation energy can effectively reduce lattice oxygen loss, thereby mitigating structural degradation associated with the generation of soluble Co species (e.g., CoO). Similarly, stronger metal‐O–O interactions enhance the structural robustness of lattice oxygen, reducing cobalt dissolution. However, these stability‐oriented modifications may compromise catalytic activity since higher oxygen vacancy formation energies can suppress the LOM pathway, while stronger metal‐O–O bonding may inhibit the dynamic surface reconstruction essential for optimal catalytic behavior. Therefore, achieving a balance between stability and catalytic performance remains a critical design consideration for next‐generation Co_3_O_4_‐based OER catalysts.

**Table** **1** summarizes the catalytic performances of recently reported spinel Co_3_O_4_‐based catalysts for acidic OER. Although considerable progress has been made in reducing their dissolution behavior, the catalytic activity of these catalysts remains significantly lower compared to noble metal‐based catalysts under similar test conditions. Therefore, it is crucial and highly beneficial to design spinel Co_3_O_4_‐based OER catalysts that are both active and stable.

**Table 1:** Summary of recently reported spinel Co_3_O_4_‐based catalysts and their catalytic activity and stability for acidic OER.

Catalyst	Electrolyte	Overpotential/mV	Stability/h	Degradation Rate/mV h^−1^	Refs.
Co_3_O_4_@C/GPO	1 m H_2_SO_4_	400@10 mA cm^−2^	40@10 mA cm^−2^	‐	[26]
TiO_2_/Co_3_O_4_	1 m H_2_SO_4_	570@10 mA cm^−2^	20@10 mA cm^−2^	2	[25b]
FTO@Co_3_O_4_/CP	0.5 m H_2_SO_4_	511@10 mA cm^−2^	21.5@10 mA cm^−2^	0.5	[25c]
Co_3_O_4_@FTO/CP	0.5 m H_2_SO_4_	646@10 mA cm^−2^	21.5@10 mA cm^−2^	0.95	[25c]
Co_3‐x_Ba_x_O_4_	0.5 m H_2_SO_4_	278@10 mA cm^−2^	110@10 mA cm^−2^	‐	[49]
p‐Co_3_O_4_	0.1 m HClO_4_	350@10 mA cm^−2^	120@10 mA cm^−2^	0.75	[29b]
Co_3_O_4‐x_F_x_	0.5 m H_2_SO_4_	349@10 mA cm^−2^	120@100 mA cm^−2^	‐	[33]
Co_3_O_4_/CeO_2_	0.5 m H_2_SO_4_	423@10 mA cm^−2^	50@10 mA cm^−2^	1.6	[35]
Mn_x_‐Co_3_O_4_	0.5 m H_2_SO_4_	431@10 mA cm^−2^	24@100 mA cm^−2^	‐	[39]
P‐Co_3_O_4_	0.1 m HClO_4_	400@10 mA cm^−2^	30@10 mA cm^−2^	1	[40]
Co_2_MnO_4_	1 m H_3_PO_4_	730@1000 mA cm^−2^	1600@200 mA cm^−2^	‐	[50]
NiMn_1.5_Co_3_O_4‐δ_	0.5 m H_2_SO_4_	280@10 mA cm^−2^	80@10 mA cm^−2^	‐	[10b]

## 4 Critical Issues in Unlocking the Structure‐Activity‐Stability Relationship

Most current research on spinel Co_3_O_4_‐based catalysts for acidic OER has focused on optimizing the catalysts’ composition, structure, and electrocatalytic performance. However, several fundamental issues remain unclear, including the identification of active sites, monitoring surface reconstructions, and other physicochemical changes during OER. A deeper understanding of these issues is crucial for guiding the development of spinel Co_3_O_4_‐based catalysts for practical applications.

### 4.1 Identifying Active Sites

Spinel Co_3_O_4_ comprises two types of geometrical Co ions with different oxidation states in its unit cell: one Co^2+^ ion in the tetrahedral site (Co^2+^_Td_) and two Co^3+^ ions in the octahedral site (Co^3+^_Oh_). The population of Co^2+^_Td_ and Co^3+^_Oh_ in spinel Co_3_O_4_ strongly influences its catalytic performance.^[^ ^13^, ^51^ ^]^ Identifying and understanding the roles of the types of Co ions is essential to improving the catalytic performance of spinel Co_3_O_4_.

**Figure 8:**
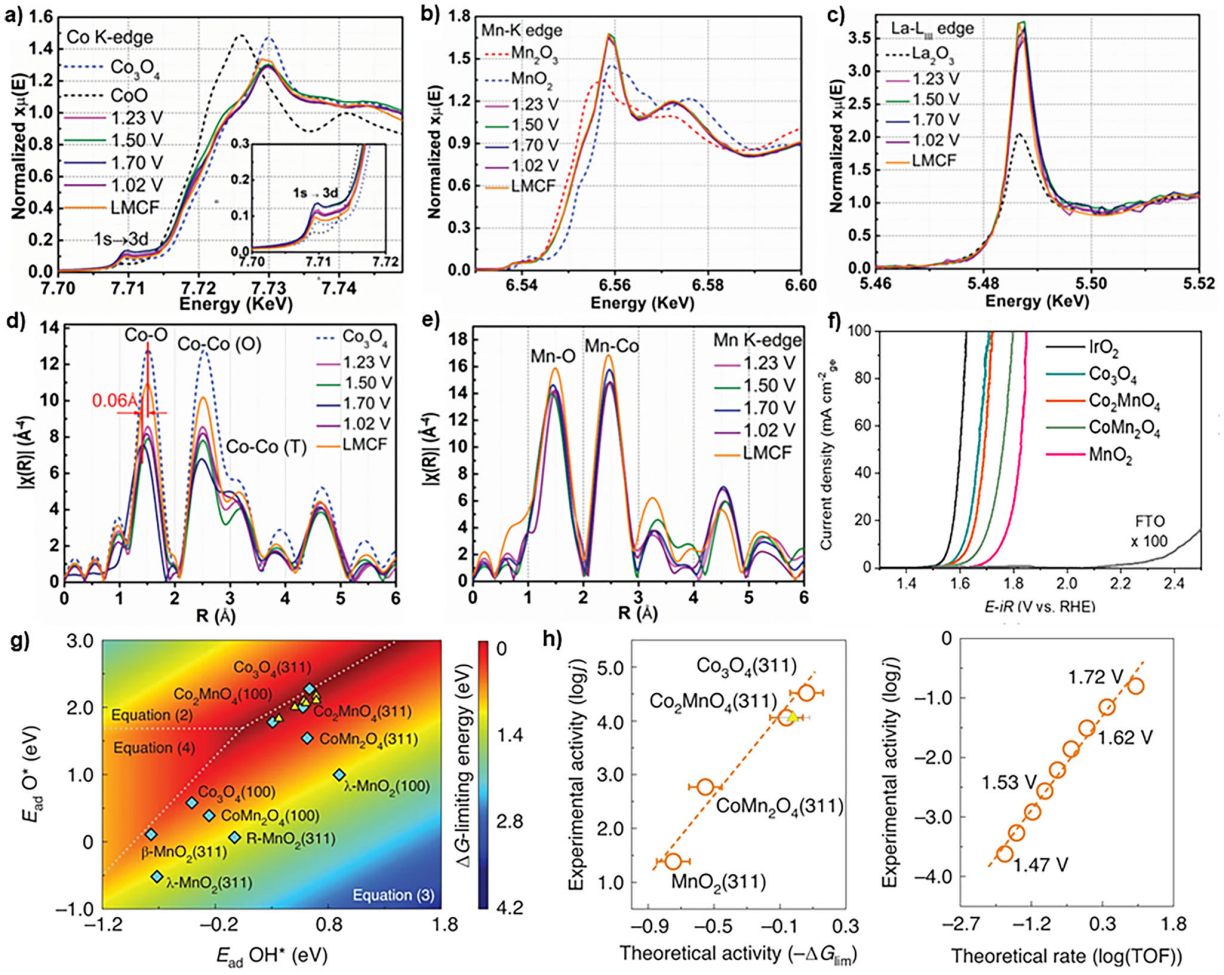
XANES spectra at a) Co K‐edge (inset: enlarged pre‐edge 1s → 3d transition), b) Mn K‐edge, and c) La L_III_‐edge of La‐ and Mn‐doped Co_3_O_4_ at different potentials. EXAFS spectra of d) Co K‐edge and e) Mn K‐edge. Reproduced with permission from ref.[53] Copyright 2023, AAAS. f) Polarization curves of Co_2_MnO_4_ and reference catalysts in 0.5 m H_2_SO_4_. g) 2D activity map with an adsorption energy of OH* as the first descriptor and adsorption energy of O* adsorption as the second descriptor. The light blue diamonds show the activity on different perfect crystal surfaces, and the yellow triangles show the activity on different defective surfaces of Co_2_MnO_4_. h) The correlation between experimental activities (log *j*) and theoretical ones (–ΔG_lim_) derived from the ΔG‐limiting energies. The comparison between the experimental current (log j) and the theoretical rate (log TOF) calculated by microkinetic modeling on Co_2_MnO_4_ at various electrode potentials. Reproduced with permission from ref.[47] Copyright 2022, Springer Nature.

In one study, Kundu et al. investigated the site‐dependent catalytic activity of Co_3_O_4_ by substituting Co^2+^_Td_ and Co^3+^_Oh_ with inactive Ti^4+^ and Cr^3+^, respectively.^[^ ^52^ ^]^ They found that Co^2+^_Td_ is essential in forming cobalt oxyhydroxide (CoOOH), which acts as a catalytically active site for OER. They concluded that Co^2+^_Td_ is more active than Co^3+^_Oh_, a finding similar to that of some other studies.^[^ ^13^, ^51^ ^]^ In another study, Cong et al. performed an in situ XAS study to investigate the active sites of Co_3_O_4_ and La‐ and Mn‐doped Co_3_O_4_ in 0.1 M HClO_4_.^[^ ^53^ ^]^ At an applied potential of 1.23 V_RHE_, their Co K‐edge X‐ray absorption near edge structure (XANES) spectra show an increased intensity in the pre‐edge 1s→3d peak, indicating a change in Co's coordination environment and electronic structure (**Figures** **8**a,d). Co's coordination number decreases, indicating a higher fraction of Co^2+^_Td_ and oxygen vacancy concentration. They also concluded that Co^2+^_Td_ serves as the catalytically active site. Further, La‐ and Mn‐doped Co_3_O_4_ display identical extended X‐ray absorption fine structure (EXAFS) spectra, indicating that La and Mn modify the structure and catalytic activity of Co^2+^_Td_ instead of directly serving as active sites (Figures 8b–e).

In contrast, several other studies reported that Co^3+^_Oh_, with a higher oxidation state, plays a dominant catalytic role while Co^2+^_Td_ is relatively catalytically inactive.^[^ ^54^ ^]^ For example, Xiao et al. demonstrated that partially substituting Co_oct_ with Mn^3+/4+^ preserved the catalytic activity of spinel Co_3_O_4_ for acidic OER while significantly improving the stability. In contrast, the complete substitution of Co^3+^_Oh_ with Mn (CoMn_2_O_4_) resulted in a substantial reduction in catalytic activity, approximately by an order of magnitude compared to pristine Co_3_O_4_. This finding suggests that Co^3+^_Oh_ in spinel Co_3_O_4_ is a key active site (Figure 8f).^[^ ^47^ ^]^ Theoretical studies revealed that Co_3_O_4_ (311) and CoMn_2_O_4_ (311) share the same RDS for the formation of *O and *OOH, whereas CoMn_2_O_4_ shows a higher energy barrier, suggesting a lower catalytic activity (Figure 8g,h).

To date, the catalytically active sites in spinel Co_3_O_4_ for OER remain under debate. The discrepancies above stem from three factors. First, differences in synthesis methods often lead to variations in catalyst morphology, which in turn result in distinct surface terminations and exposed crystallographic facets.^[^ ^55^ ^]^ For example, hexagonal‐phase Co_3_O_4_ (h‐Co_3_O_4_) with mixed {111} and {110} facets has demonstrated enhanced OER activity, primarily due to the greater presence of Co^3+^ in octahedral coordination.^[^ ^56^ ^]^ However, this increased Co^3+^ content has also been associated with catalyst degradation over time, owing to the formation of soluble CoO_2_ species. The partial substitution of octahedral Co by Ru has proven effective in mitigating Co dissolution while creating highly active catalytic sites. Second, elemental doping plays a crucial role in modulating the local electronic structure and coordination environment of Co sites.^[^ ^57^ ^]^ Such modifications can be accompanied by the formation of lattice defects that switch reaction pathways for stability enhancement.^[^ ^28^, ^58^ ^]^ Third, under electrochemical operation, particularly at anodic potentials relevant to OER, the catalyst surface undergoes significant structural and chemical transformations to form new catalytically active species, such as Co^(IV)^═O and Co^(III)^─OH.^[^ ^13^, ^30^ ^]^ These potential‐dependent evolutions introduce additional complexity, making it challenging to attribute activity to a specific Co site. Collectively, these factors underscore the inherent complexity of identifying the true active sites in Co_3_O_4_‐based catalysts and highlight the necessity of employing operando and in situ characterization techniques to monitor their dynamic behavior under reaction conditions.

### 4.2 Monitoring Surface Reconstruction

**Figure 9:**
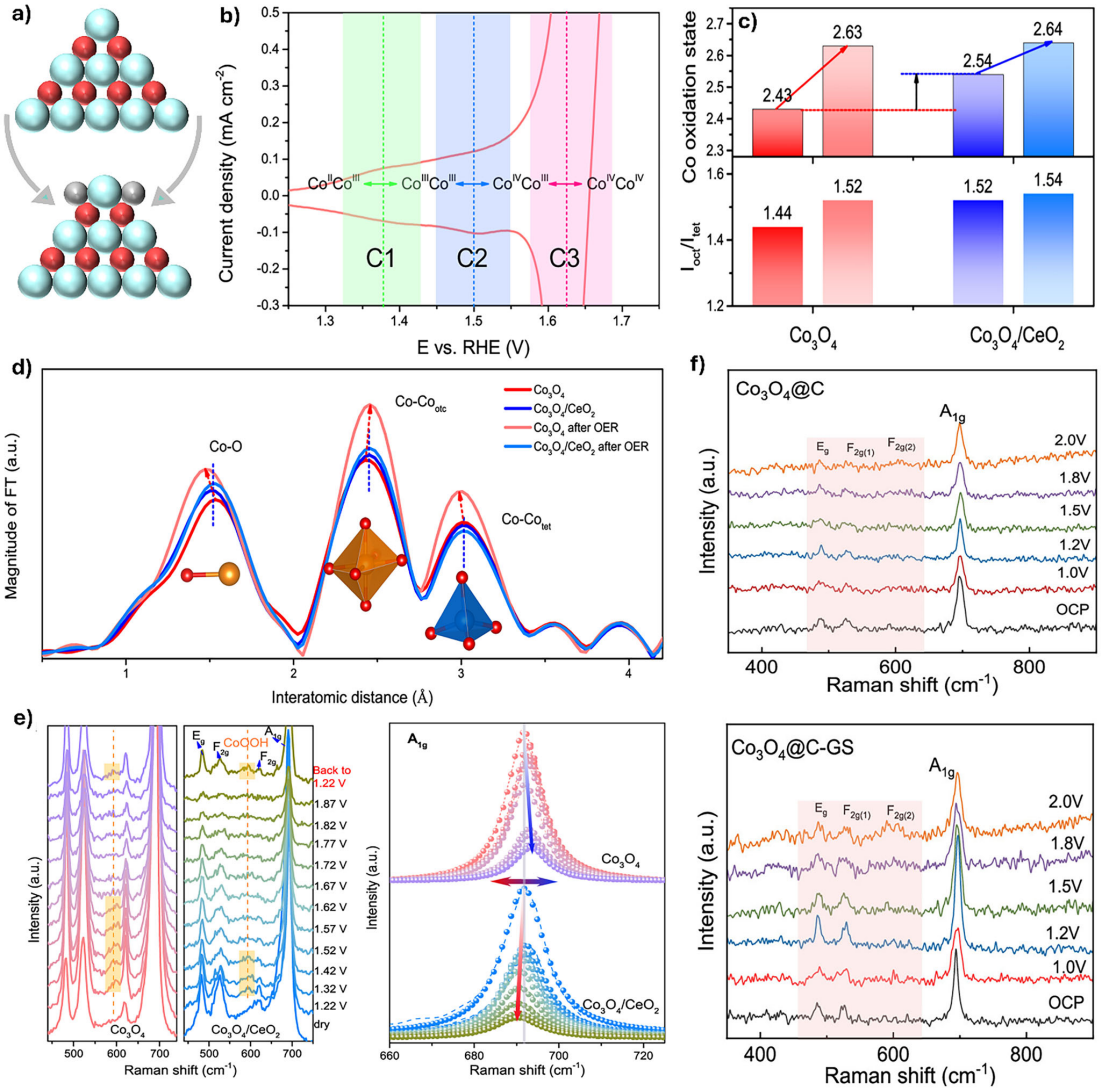
a) Schematic illustration of the phase change of Co_3_O_4_‐based catalyst. b) CV curve of Co_3_O_4_ catalyst in 0.5 m H_2_SO_4_. c) the average Co oxidation states and the intensity ratios of Io_ct_/I_tet_ (oct: octahedral Co site; tet: tetrahedral Co site) of Co_3_O_4_ and Co_3_O_4_/CeO_2_. d) FT‐EXAFS of Co K‐edge spectra before and after OER, e) in situ Raman spectra of Co_3_O_4_ and Co_3_O_4_/CeO_2_ at various potentials (left panel) and the Raman A_1g_ peaks of Co_3_O_4_ (top) and Co_3_O_4_/CeO_2_ (bottom) (right panel). Reproduced with permission from ref.[30a] Copyright 2021, Springer Nature. In situ Raman spectra of f) Co_3_O_4_@C and Co_3_O_4_@C‐GS at various potentials. Reproduced with permission from ref.[25a] Copyright 2023, ACS.

Metal oxide‐based catalysts typically undergo surface reconstruction under an applied potential during OER (**Figure** **9**a). Monitoring catalyst surface reconstruction offers valuable insights into the interaction between reactants and catalysts, as well as the underlying reasons for varied catalytic activity and stability. Recent studies have tried to study the surface reconstruction of spinel Co_3_O_4_ during OER.

For example, Huang et al. investigated the structural evolution of spinel Co_3_O_4_ during acidic OER.^[^ ^30a^ ^]^ Three distinct pre‐OER redox features were observed in the cyclic voltammetry (CV) curves in 0.5 m H_2_SO_4_, with the cathodic peaks (C1, C2, and C3) attributed to the structural evolution process: Co^2+^Co^3+^ ↔ Co^3+^Co^3+^ ↔ Co^3+^Co^4+^ ↔ Co^4+^Co^4+^ (Figure 9b). Ex situ XAS reveals significant changes in the bond distances in Co_3_O_4_ after OER, including decreased Co‐O and Co‐Co_tet_ bonds and increased Co‐Co_oct_ bonds (Figure 9c,d). Additionally, an increase in the I_oct_/I_tet_ (oct: octahedral Co site; tet: tetrahedral Co site) ratio from 1.44 to 1.52 after OER indicates notable changes in the crystal structure of Co_3_O_4_ (Figure 9c). The in situ Raman spectroscopy further proves a phase transformation process, where a new Raman signal at ≈600 cm^−1^ at 1.22 V_RHE_ is ascribed to the formation of surface CoOOH species (Figure 9e). Importantly, the pre‐redox behavior of Co_3_O_4_ can be effectively regulated by the introduction of CeO_2_. On the other hand, the bond distances in the Co_3_O_4_/CeO_2_ and Io_ct_/I_tet_ ratios are almost identical before and after OER. On the other hand, the incorporation of CeO_2_ facilitates the oxidation of active Co sites to Co^4+^ species, which serve as active centers, thereby enhancing the OER kinetics and improving the catalytic activity of Co_3_O_4_/CeO_2_.

Additionally, monitoring the dynamic structure evolution of spinel Co_3_O_4_‐based catalysts during the OER process is essential to improve their stability performance.^[^ ^8^, ^59^ ^]^ Liu et al. applied operando Raman to investigate the surface structure evolution of Co_3_O_4_@carbon (Co_3_O_4_@C) and Co_3_O_4_@carbon on graphite sheets (Co_3_O_4_@C‐GS) during acidic OER. The Raman peaks at 480, 520, and 690 cm^−1^ correspond to the E_g_, F_2g_, and A_1g_ modes for Co_3_O_4_, respectively (Figure 9f). Compared to Co_3_O_4_@C‐GS, the peaks located at 480 and 520 cm^−1^ of Co_3_O_4_ @C gradually disappear with increased applied potentials, suggesting an unstable local bonding environment of Co_3_O_4_@C during OER. In contrast, these peaks of Co_3_O_4_@C‐GS are well‐maintained. These results indicate that graphite sheets enhance the OER activity of Co_3_O_4_@C by accelerating interface charge transfer and promoting catalytic stability through the construction of a hierarchical interface architecture.

### 4.3 Tracking Degradation Pathways

**Figure 10:**
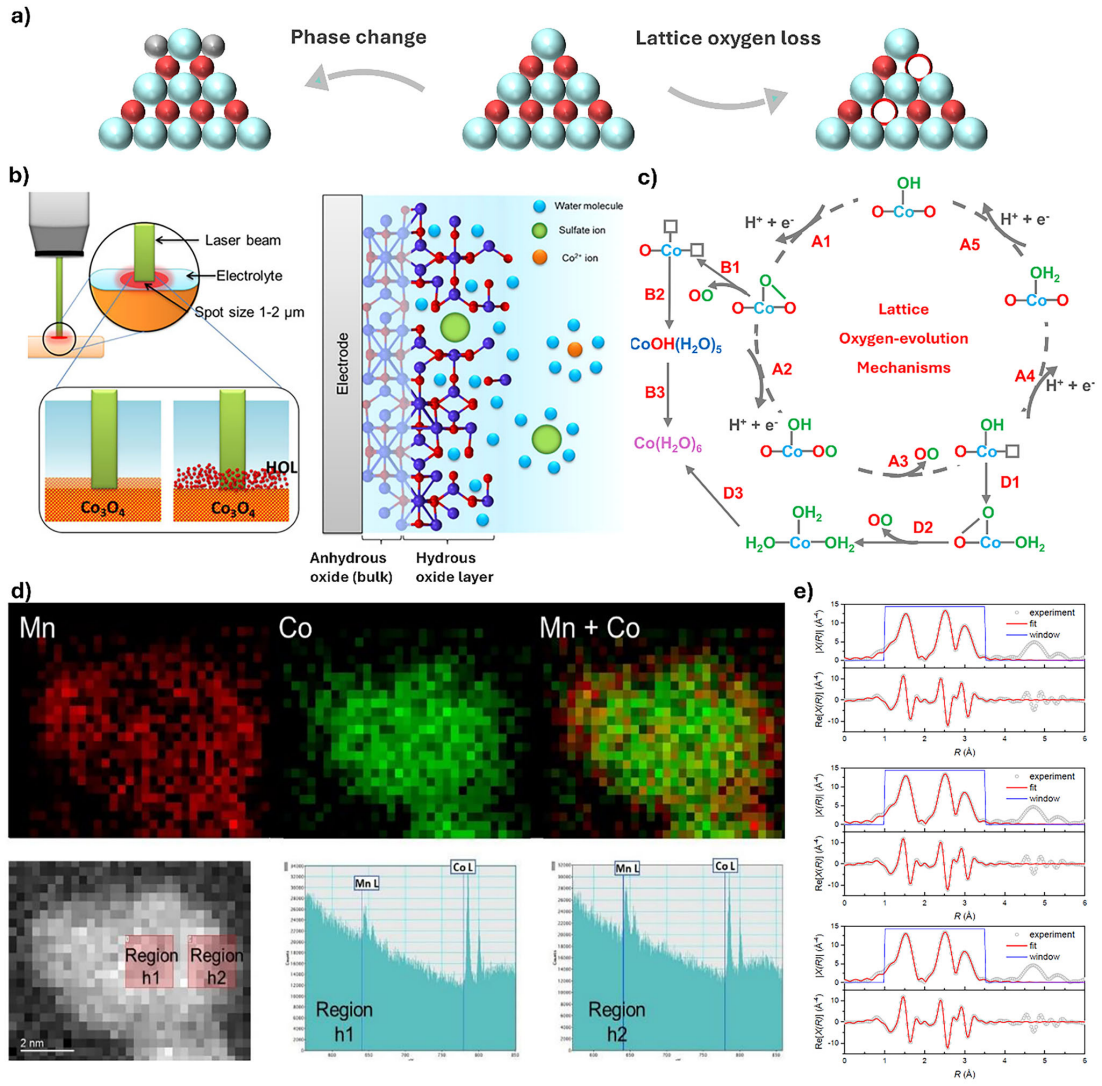
a) Schematic illustration of the degradation pathways of Co_3_O_4_‐based catalyst. b) in situ Raman of SCoC‐700, full width at half‐maximum of A_1g_ mode in SCoC‐700 at different times, and c) proposed lattice oxygen mechanism of Co_3_O_4_. Reproduced with permission from ref.[60] Copyright 2021, ACS. d) STEM‐EELS analysis of Co_2_MnO_4_ after electrolysis at a current density of 100 mA cm^−2^ for 23 h in 1 m H_2_SO_4_. e) Fitting analysis of Fourier‐transformed k^3^‐weighted EXAFS spectra of Co K‐edge before and after electrolysis for 4 and 23 h at a current density of 100 mA cm^−2^ in 1 m H_2_SO_4_. Reproduced with permission from ref.[47] Copyright 2022, Springer Nature.

The poor stability of spinel Co_3_O_4_‐based catalysts in acidic OER is also observed to correlate with various structural changes (**Figure** **10**a). Although Co_3_O_4_‐based catalysts are considered theoretically stable under acidic conditions, they tend to form high‐valence state soluble Co^4+^ species at high applied potentials, leading to structural degradation. Consequently, understanding the underlying details of these degradation pathways under OER operating conditions is essential for controlling them and eventually improving the stability of catalysts.

Some studies have focused on the valence state changes of Co_3_O_4_‐based catalysts during degradation. For example, Natarajan et al. explored the origin of Co_3_O_4_ dissolution by operando Raman and CV.^[^ ^60^ ^]^ Angle‐resolved XPS and theoretical calculations revealed that the ionocovalent CoO_4_ species promote the acid stability, while the covalent CoO_6_ facilitates lattice oxygen evolution and structural degradation. At an anodic potential, the generation of a CoO_2_ intermediate and the loss of lattice O result in undercoordinated CoO sites that react with water, forming an amorphous hydrous oxide layer of CoO(OH)_x_ (Figure 10b). Due to the involvement of lattice O in the LOM pathway, the remaining Co cations readily dissolve in the electrolyte and then diffuse away from the electrode, which is simultaneously oxidized to higher valence states with soluble characteristics (Figure 10c).^[^ ^61^ ^]^ This indicates that the formation of lattice O evolution and O vacancies induced by the CoO_6_ species promotes OER activity but reduces stability. In another study, Li et al. also investigated the dissolution behavior of Co_2_MnO_4_ for OER in acidic conditions.^[^ ^47^ ^]^ EELS and inductively coupled plasma mass spectrometry results indicate that Co leaching is more pronounced than that of Mn counterparts during the stability test (Figure 10d). Importantly, Rietveld refinements and EXAFS spectra fitting results suggest that Co^2+^_Td_ sites are readily dissolved (Figure 10e). Notably, the preferential leaching of specific Co species (e.g., Co^2+^_Td_ sites) underscores the importance of tailoring local atomic configurations for enhanced stability. For example, incorporating dopants or forming multi‐metal oxides with elements like Mn, Ti, or Cr to stabilize specific coordination sites, thus suppressing dissolution and enhancing catalytic performance.

## 5 Conclusion and Perspective

The sluggish OER kinetics and high cost of noble metal‐based catalysts have hindered the widespread deployment of certain critical energy conversion and storage technologies, including PEM water electrolyzers, CO_2_ and N_2_ electrolyzers, and metal‐air batteries. Spinel Co_3_O_4_‐based catalysts, which have theoretical catalytic activity comparable to that of noble metal‐based catalysts, face challenges due to their poor stability in acidic electrolytes, compromising their operational stability.

This review begins by exploring OER mechanisms and then systematically summarizes recent research studies on improving the stability of Co_3_O_4_‐based catalysts in acidic electrolytes. Afterward, research studies on elucidating the structure‐activity‐stability relationship using operando characterization techniques are discussed, focusing on identifying active sites, monitoring surface reconstruction, and tracking degradation pathways. In our view, achieving practical applications of spinel Co_3_O_4_‐based OER catalysts requires a more comprehensive understanding of their reaction mechanisms, utilizing advanced characterization tools to optimize the atomic structures of active sites and the physical structures of catalysts. This also involves developing new catalyst development methodologies and evaluating catalysts under industrially relevant conditions. We further discuss these issues in detail below.

### 5.1 Advanced Characterization Tools

Spinel Co_3_O_4_‐based catalysts commonly undergo surface reconstructions during OER, and these surface sites are often closely related to their catalytic performance. Thus, probing these dynamic surface changes using in situ/operando characterization techniques can help comprehensively understand OER mechanisms on these sites. However, current in situ/operando characterization techniques, such as XAS and Raman spectroscopy, require several minutes to hours to acquire a spectrum, limiting their ability to capture transient states in catalysts. They often detect only (quasi)stable active sites under applied potentials, which might not represent real RDSs. Moreover, reaction intermediates typically have lifetimes in the order of picoseconds, necessitating the development of fast‐speed spectroscopic techniques to monitor dynamic structural and oxidation state changes of active sites induced by reaction intermediates. Further, isotope labeling can also be used to investigate chemical bond breaking and formation involving oxygen‐containing intermediates during OER. These technical gaps necessitate rapid in situ/operando characterization techniques, enabling the determination of structures of active sites, reaction intermediates, and reaction pathways under reaction conditions with greater accuracy. The new insights gained from these characterization techniques can provide a deeper understanding of OER mechanisms and their correlation with local structures in spinel Co_3_O_4_‐based catalysts, guiding the design and enabling more precise control of active sites in catalysts.

### 5.2 Atomic Structures of Active Sites

The exact catalytically active sites in spinel Co_3_O_4_‐based catalysts during OER are still under debate. Currently, the selective substitution of Co^3+^_Oh_ and Co^2+^_Td_ with heteroatoms has been a key approach to influencing the active sites of catalysts. Substituting cations would create different coordination environments in Co_3_O_4_. In particular, two types of active site structural optimization are essential. First, different substitution elements have different effects on catalytic activity and stability. For example, Ni and Fe enhance catalytic activity, while Mn, Ti, and Cr form more stable M─O bonds, improving stability. Balancing activity and stability enhancement requires the optimization of substitute elements. Second, the locations (e.g., surface vs bulk vs specific crystal facets) of substitute elements in Co_3_O_4_ can strongly influence the creation of desired active site atomic structures. Thus, precisely controlling the locations of substitute elements is also critical. In this regard, advanced structural characterization tools, such as TEM with a sub‐angstrom resolution, are crucial to provide detailed local crystal structures and guide the design of active sites. Alternatively, multi‐metal oxides exhibit unique characteristics, including strains, coordination environments, and ligand effects. For instance, certain late transition metal oxides, such as TiO_2_, SnO_2_, Ta_2_O_5_, and Nb_2_O_5_, exhibit excellent stability but limited catalytic activity for acidic OER. Thus, forming multi‐metal oxides between these transition metal oxides and Co_3_O_4_ may also change the atomic structures of active sites and yield higher stability.

### 5.3 Physical Structures of Catalysts

The physical structure of spinel Co_3_O_4_ significantly influences its stability, which can be optimized through various approaches. First, the surface of spinel Co_3_O_4_ catalysts can be engineered through suitable pre‐treatment or electrochemical cycling, thereby enhancing surface lattice oxygen activity while maintaining the bulk structure's integrity. In particular, a practical approach involves constructing spinel Co_3_O_4_ catalysts with a core‐shell architecture, in which the active shell enhances lattice oxygen participation during the OER, while the structurally stable core provides mechanical robustness. Second, constructing surface passivation layers or coatings on spinel Co_3_O_4_ can mitigate excessive lattice oxygen release. As discussed in Section 3.1, stable oxides (e.g., TiO_2_) can be used to construct surface layers. Third, spinel Co_3_O_4_ catalysts often suffer agglomeration and detachment from electrode substrates under applied potentials. Anchoring Co_3_O_4_ on suitable support materials may enhance its stability. Common support materials include carbon materials, Ti foil, and fluorine‐doped tin oxide (FTO) glass. Ti foil and FTO glass have higher oxidation resistance. However, metallic Ti is prone to be oxidized into TiO_2_ during OER, resulting in low electrical conductivity. Some studies have explored doping V or Nb into Ti to prevent oxidation of Ti.^[^ ^62^ ^]^ Fourth, creating spinel Co_3_O_4_ catalysts with porous structures, such as nanoarrays, can be explored to expose active sites and facilitate the release of oxygen bubbles, thereby enhancing their structural stability.

### 5.4 Catalyst Development Methodology

Current catalyst development is typically preceded by trial and error, a process that is both time‐consuming and costly. Various theoretical tools should be used to improve catalyst development efficiency. For example, Pourbaix diagrams can be used to identify acid‐stable metal/metal oxide phases, allowing for improved catalyst stability. New machine learning and artificial intelligence tools can account for multi‐parameter systems in complex and dynamic reaction environments while operating with reduced computational costs. They should be used to speed up catalyst discovery, design, and synthesis.

### 5.5 Stability Evaluation at Industry‐Relevant Conditions

Although spinel Co_3_O_4_‐based catalysts have demonstrated promising stability under mild laboratory conditions, i.e., ambient temperature, low current densities (≤10–100 mA cm^−2^), small electrode geometries, and diluted electrolytes, which differ markedly from the operating conditions of industrial water electrolyzers. Under industrial‐relevant conditions, poor long‐term catalyst stability and interfacial incompatibility with ionomers, particularly under sustained high current and voltage conditions, hinder their practical adoption. Bridging this gap necessitates rigorous performance evaluations under industrial‐relevant conditions, including testing membrane electrode assemblies at high current densities (e.g., 1–3 A cm^−2^), elevated temperatures (60–80 °C), and continuous reactant flow with dynamic gas bubble evolution. Additionally, the use of tap water as a water feedstock is recommended, as it provides insights into the resilience of catalysts against common impurities in water, such as chloride, sulfate, and various organic contaminants, which can strongly affect decentralized and resource‐limited hydrogen production applications. Given the impracticality of assessing catalysts’ durability over their entire operational lifetime (e.g., >100,000 h), accelerated stress tests (ASTs) are recommended to evaluate long‐term stability within experimentally feasible time scales. AST protocols can simulate long‐term degradation under stress conditions, such as alternating current cycling, open‐circuit voltage exposure, and rapid current density modulation, enabling the rapid identification of degradation mechanisms and thus helping the rational design of catalysts. Furthermore, it is also helpful to adopt standardized and quantitative stability metrics and report such findings. These may include, but are not limited to, metal dissolution rates (e.g., µg cm^−2^ h^−1^), voltage degradation rates (e.g., mV h^−1^), and details of MEA structures and their performance parameters (e.g., cell efficiency, materials cost). Research findings based on detailed and industry‐relevant metrics will enable fair benchmarks among various studies and provide more accurate assessments for catalysts’ practical viability.

## Conflict of interest

The authors declare no conflict of interest.
